# Timing and Tuning for Familiarity of Cortical Responses to Faces

**DOI:** 10.1371/journal.pone.0076100

**Published:** 2013-10-09

**Authors:** Maria A. Bobes, Agustin Lage Castellanos, Ileana Quiñones, Lorna García, Mitchell Valdes-Sosa

**Affiliations:** 1 Cognitive Neuroscience Dept., Cuban Neurosciences Center, Havana, Cuba; 2 Basque Center on Cognition, Brain and Language (BCBL), Donostia, Spain; University of Leuven, Belgium

## Abstract

Different kinds of known faces activate brain areas to dissimilar degrees. However, the tuning to type of knowledge, and the temporal course of activation, of each area have not been well characterized. Here we measured, with functional magnetic resonance imaging, brain activity elicited by unfamiliar, visually familiar, and personally-familiar faces. We assessed response amplitude and duration using flexible hemodynamic response functions, as well as the tuning to face type, of regions within the face processing system. Core face processing areas (occipital and fusiform face areas) responded to all types of faces with only small differences in amplitude and duration. In contrast, most areas of the extended face processing system (medial orbito-frontal, anterior and posterior cingulate) had weak responses to unfamiliar and visually-familiar faces, but were highly tuned and exhibited prolonged responses to personally-familiar faces. This indicates that the neural processing of different types of familiar faces not only differs in degree, but is probably mediated by qualitatively distinct mechanisms.

## Introduction

All known faces are charged with significance. However, the face of a slightly known next-block neighbor is not equivalent to that of a spouse. The former can provoke a flicker of recognition, whereas the latter induces longer lasting affective states. Different kinds of known faces differ in the amount and quality of information that they afford, and probably in the neural systems that process them. Accordingly, faces that rally different types of memory will activate cortical areas differentially. These effects vary according to which areas of the face processing system are involved. The face processing system is divided in the core face processing areas – comprised of occipital face area (OFA), fusiform area (FFA) and posterior superior temporal sulcus (pSTS) – and the extended face processing system (e.g. posterior cingulate (PC), temporal cortices, anterior cingulate (AC) and middle orbitofrontal cortex (mOF)) [1], [2].

Several fMRI studies convincingly show a differential involvement of the face-responsive cortical regions in the processing of distinct types of familiarity. In these studies faces of famous or personally significant people, and also visually familiar faces served as stimuli [3], [4]. Nevertheless, more information is needed to fully characterize the role of these brain areas in processing face related memories. All of the cited studies focus on the comparison of responses to pairs of face types. Furthermore, only a few studies have examined responses to more than one type of known face in the same subjects. Therefore, it is difficult to infer the selectivity of each area across a range of face-related memories. What is needed is an activation-profile analysis (see [5]), where the relative strength of the response to each type of face can be compared in the same individuals. This is analogous (in the memory domain) to the tuning curves measured for different values of stimulus parameters. Tuning for lower order properties of faces (such as viewpoint or geometrical properties) has been measured for the core face areas [6] [7], but not for different types of face associated memories. Tuning for face related properties has not been studied within the extended face system.

At the same time, it is likely that different types of familiar faces not only involve distinct neural systems, but also are associated with different temporal courses of processing. Measurements of the time course of functional magnetic resonance imaging (fMRI) hemodynamic-responses to pictures or words (using flexible hemodynamic response function (HRF) models that can vary in shape) have revealed significantly longer durations for emotionally charged than for neutral stimuli [8], [9]. The fact that the increased duration can be measured (despite the poor time resolution of fMRI) is an indication that the emotional processes involved are effectively persistent in time. However, the duration of responses to faces with different types of familiarity (i.e face of acquaintances/celebrities versus unfamiliar faces) has not been compared to date, despite their obvious difference in socio-emotional content.

Here we address the two issues introduced above by comparing the fMRI responses to faces of close personal acquaintances, artificially learned faces (associated only with visual memories), and unfamiliar faces, within the same group of subjects. The temporal course of the hemodynamic responses (estimated with multi-parameter HRF models), and their tuning for different types of faces, were measured in regions of interest within both the core and extended face processing system. We hypothesized that the tuning would become more specific and response duration would increase as one progresses along the ventral visual pathway into frontal areas belonging to the extended face system. To anticipate the results, these predictions were partially verified: we found little tuning and short responses in the core areas, with the most extreme tuning (i.e. exclusive sensitivity to personally familiar faces) and the longest responses in the mOF and AC cortex.

## Methods and Materials

### Participants

Participants were screened to exclude neurological, psychiatric, and systemic diseases, and then recruited as volunteers. The sample is comprised of ten right-handed healthy subjects (5 males and 5 females) that participated in the experiment as non-paid volunteers. Their ages ranged from 21 to 36 years (mean = 27.9), all had university degrees. Participants provided written informed consent to participate in this study, which were documented in forms signed by participants. The experimental protocols were approved by the Ethics Committee of The Cuban Neurosciences Center.

### Stimuli

Stimuli consisted of photographs of familiar and unfamiliar faces, and of houses. Two different sets of familiar faces were obtained: Acquaintance faces, which were selected among the family and close friends of each subject (i.e. these were different across participants); and newly-learned faces, which were previously unknown faces, learned after exhaustive exposure in the lab (see below). Although these two types of faces stimuli were both visually familiar, only the acquaintances possessed social-emotional significance for each participant.

Black and white photographs of the faces in frontal views were digitally processed (to minimize differences in size, contrast, and overall luminance) and displayed within a circular border masking external facial-features, clothes, and the background.

### Training for newly-learned faces

Learning took place during six sessions (two sessions per day) two weeks before the fMRI session. Each session consists of a study and a test period. In the study period, subjects viewed the set of 15 initially unknown faces (the same in all sessions) on a CRT monitor in a random order, with the onset and offset times under the subject’s control. The test period consisted of a familiarity decision task. The 15 studied (“old”) faces were presented interspersed with 15 “new” unfamiliar faces, one at a time in a random order. The new unfamiliar faces were never repeated. The subjects verbally classified each face as old or new, and were provided feedback on the accuracy of the response. The faces in the training set were thus seen twelve times, after which subjects reached more than 98% of accuracy. Before the recording session, subjects viewed once again the learned faces randomly mixed with new unfamiliar faces.

### Image acquisition

A Siemens 1.5T Magnetom Symphony system with a standard birdcage head coil for signal transmission/reception (Siemens, Erlangen, Germany) was used to acquire all images. BOLD-contrast-weighted echoplanar images for functional scans consisted of 16, interleaved, axial slices of 5 mm thickness (no interslice skip) that partially covered the brain from about −40 below to about 40 mm above the AC-PC plane. In-plane resolution was 1.73×1.73 mm, with the following parameters: FOV = 512×512 mm; matrix = 128×128; echo time (TE) = 60 ms; TR = 2 s with no time gap; flip angle = 90 degrees. The first five volumes of each run were discarded to allow for T1 equilibration effects. Subsequently, a MPRAGE T1-weighted structural image (1×1×1 mm resolution) was acquired for coregistration and display of the functional data, with the following parameters: echo time (TE) = 3930 ms, repetition time (TR) = 3000 ms, flip angle = 15 degrees, and field of view (FOV) = 256×256×160 mm^3^. This yielded 160 contiguous 1 mm thick slices in a sagittal orientation.

### fMRI procedure

Participants were presented with four sets of stimuli: 15 houses, 30 unfamiliar faces, 15 faces of acquaintances, and 15 newly-learned faces. Within the MRI scanner, the participants viewed the stimuli in a pseudo-random order, which were presented for 1000 ms, with random inter-stimulus intervals (ISI) varying between 4000 and 6000 ms. The complete set of stimuli was repeated in three runs, each time in a different random order, separated by 1 minute breaks. Different unfamiliar faces were used in each run. Subjects were required to silently count any familiar faces. Runs lasted about 7.46 min. Stimuli were rear-projected onto the center of an opaque screen located at the subject’s feet and viewed with a mirror fixed to the head coil.

### Functional data analysis

Functional data were analyzed using SPM5 and related toolboxes (Wellcome Department of Imaging Neuroscience; http://www.fil.ion.ucl.ac.uk/spm). Outlier functional scans and slices were repaired with the Artifact Repair Toolbox, (Gabrieli Cognitive NeuroScience Lab; http://cibsr.stanford.edu/tools/ArtRepair/ArtRepair.htm), after which the images were slice-time corrected taking the middle slice as reference (using SPM5′s phase shift interpolation with the unwarp option) and then realigned to the first image in the session. The anatomical T1 image was coregistered [10] with the whole brain EPI, which in turn was coregistered with the mean of these limited field-of-view EPIs. Each participant’s T1 scan was bias corrected, then spatially normalized to MNI-space and segmented into gray matter (GM), white matter (WM), and cerebrospinal fluid using the unified procedure in SPM5 [11]. The parameters for normalization of the anatomical image were used to transform the functional scans to MNI space. Normalized images were spatially smoothed using an 8 mm Gaussian kernel. Data were high-pass filtered (64 s cut-off period).

A massive univariate general linear model (GLM) was applied, using for each stimulus category a set of design covariates that was obtained by convolving the canonical hemodynamic response function, plus time and dispersion derivatives (provided by SPM5), with delta functions located at stimulus onsets [12]. Also the six motion-correction parameters were included in the design matrix. Parameters of the GLM were estimated with a robust regression using weighted-least-squares, which was corrected for temporal autocorrelation in the data (Diedrichsen&Shadmehr; http://www.bangor.ac.uk/~pss412/imaging/robustWLS.html). A t-statistic was then obtained for each voxel for the contrast of interest in each subject.

Unfamiliar faces were divided in two subsets for the analysis. A subset of 15 unfamiliar faces were used for the contrast unfamiliar faces >houses. This contrast was used as a functional localizer of face selective areas. A different subset of unfamiliar faces (the remaining 15 unfamiliar faces) was used to estimate the remaining contrasts of interest: acquaintances -faces>unfamiliar faces, newly-learned faces>unfamiliar faces and acquaintances faces> unfamiliar faces. The two groups of unfamiliar faces were randomly selected from the three experimental runs. Consistent effects across subjects were tested employing the SPM5 random effects model, in which the contrast images for all the subjects were entered. The threshold for this analysis was set at p<0.005 (uncorrected).

Region of interest (ROIs): Functional regions of interest were obtained in different ways for core and extended system areas. For the core system ROIs were obtained directly from the functional localizer contrast unfamiliar faces >houses, using a relaxed threshold of p<0.05 (uncorrected) to obtain larger ROIs. This contrast evinced activation in bilateral OFA, FFA and right pSTS. However, this contrast did not uncover extended system areas since these areas present weak or no response to unfamiliar faces.

The extended system ROIs were obtained starting with preliminary search regions extracted from a previous study using a similar paradigm [13]. These regions were located in the following areas of the AAL atlas [14]: PC, AC, mOF, inferior frontal pars triangularis (FrI), left insula (Ins), anterior temporal (AT) and hippocampus. Next, these regions were defined using the results of a contrast analysis with our experimental data. To avoid circularity, this contrast analysis was performed with a leave-one-subject-out procedure (LOSO) [15]. A single subject was iteratively left out and a second level random effect analysis was performed for the contrast all faces >houses and the significant voxels were intersected with the previously defined search regions.

A total of 14 ROIs were obtained: five from the unfamiliar faces>houses contrast (left and right OFA, left and right FFA, and right pSTS) and nine from all faces>houses contrast with the LOSO procedure (left and right PC, left and right AC, left and right mOF, left and right FrI and left Ins). AT and hippocampus were not included due to the inconsistency of the response in these areas (only 7 subjects present activation at the AT area and 5 at the hippocampus).

### Estimation of HRF

The spatially unsmoothed time series in the ROIs were averaged over voxels obtaining one time series for each ROI and for each run. To estimate the hemodynamic response the same design matrix described before was used. The corresponding coefficients for each column were obtained using linear regression. After the coefficients were obtained the hemodynamic responses waveforms were calculated as the linear combination of the three basic functions scaled by their respective coefficients.

Extraction of HRF parameters: Three parameters were extracted from the heamodynamic response waveform: height, latency and width. They were extracted after fitting a Gaussian function to the obtained HRF. The amplitude was defined as the maximum of the Gaussian function, and the latency corresponds to the location of this maximum. Accordingly, the width of the response was defined as the standard deviation of the fitted Gaussian. The area of the HRF was defined as the area of the fitted Gaussian, multiplied by the sign of the peak. The height of each face condition in each ROI was referred to the house response by subtraction. Repeated measured analysis of variance (rm ANOVA) were preformed independently over the log transformation of height, latency and width parameters estimated for each face stimulus, using two main factors: face category and ROI. Significant level for planned comparison was set at p<0.05. When appropriate, the Huynh-Feldt correction [16] was used to mitigate violations of the sphericity assumptions in the repeated measures ANOVA (the corresponding epsilon values are reported).

## Results

All participants counted the familiar faces (acquaintance and newly-learned faces) accurately: >95% correct for every subject. After the fMRI scanning session, subjects were asked to rate the affective valence of each familiar person using a 5-point likert scale: 1- “very unpleasant”, 2- “unpleasant”, 3- “neutral”, 4- “pleasant”, 5- “very pleasant”. The mean ratings reached for faces of acquaintances were 4.31 (SD = 0.18) – thus dominantly judged as pleasant – and for newly-learned were 2.81 (SD = 0.25) – near neutral. The faces of acquaintances were judged more pleasant than the merely visually familiar newly-learned faces (t = 10.69, df = 9, p<0.0001).

### Voxelwise analysis

**Figure 1:**
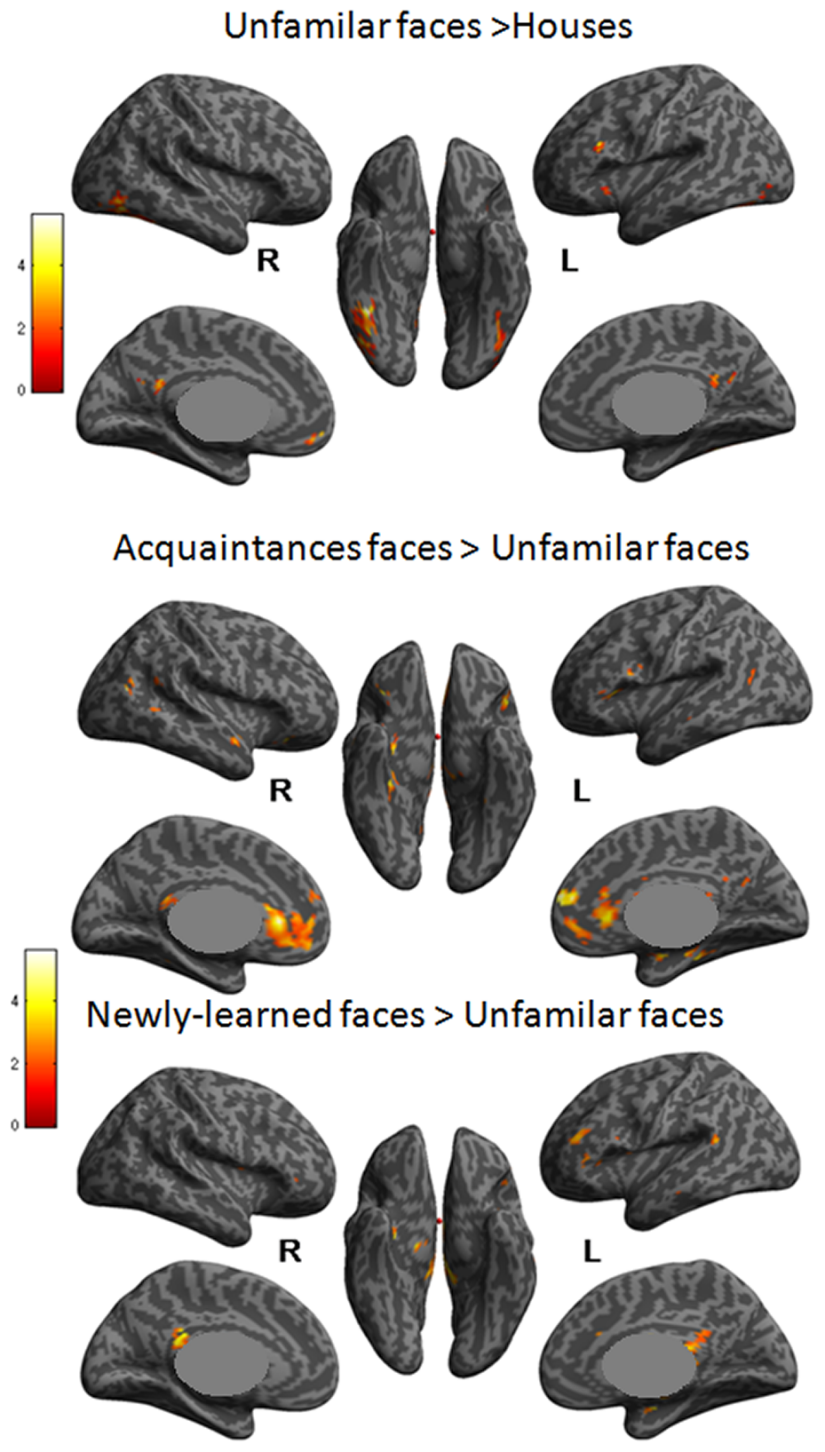
Face-related activation found in the voxelwise analysis. Activation maps indicate regions where the response was higher for: A) unfamiliar faces than houses. B)acquaintance faces than unfamiliar faces. C) newly-learned faces than unfamiliar faces. These activations are shown on an inflated brain, depicting voxels surviving p<.005 (uncorrected).

In order to localize the brain areas responding to different face conditions, a second level random effects analysis over three different contrasts was carried out (Figure 1). Face selective areas were defined by the contrast of unfamiliar faces>houses for each subject. (For these comparisons we used a subset of unfamiliar face not used further for the HRF estimations.) The results of the random effects model for this contrast are shown in Figure 1A and Table 1. Two main clusters of activation were found bilaterally, which correspond to the FFA and OFA. Some other areas, PC, mOF, and left FrI, also exhibited significant activations (Figure 1A, Table 1). Brain areas related to face familiarity processing were located by analyzing the contrasts: acquaintances>unfamiliar faces and newly-learned>unfamiliar faces (Figure 1B, C). The responses to faces of acquaintances were larger than the responses to unfamiliar faces in several regions, including AC, mOF, PC, left FrI, left and right pSTS, right anterior FFA, and the right parahippocampus (Figure 1B). Smaller clusters also appeared in other regions (Table 2). However, the cortical response to newly-learned faces was more limited, evoking larger BOLD responses than unfamiliar faces only in PC, left middle temporal, right hippocampus and the left FrI (Figure 1C, Table 3).

**Table 1:** Clusters of face-selective activations in a Second level random-effects analysis for the localizer contrast (unfamiliar faces>houses).

cluster p(cor)	cluster equiv k	voxel p(FDRcor)	voxel T	voxel p(unc)	x	y	z	
0.157	299	1.000	5.63	0.000	38	−46	−22	Fusiform_R
		1.000	4.89	0.000	44	−64	−14	Occipital_Inf_R
0.618	164	1.000	5.24	0.000	2	−58	22	Precuneus_R
		1.000	4.44	0.001	−4	−48	22	Cingulum_Post_L
		1.000	4.02	0.002	4	−48	20	Precuneus_R
1.000	33	1.000	4.49	0.001	−42	20	20	Frontal_Inf_Tri_L
0.988	70	1.000	3.96	0.002	−40	−52	−16	Fusiform_L
		1.000	3.61	0.003	−42	−74	−12	Occipital_Inf_L
1.000	25	1.000	3.67	0.003	−6	52	−8	Frontal_Med_Orb_L
1.000	7	1.000	3.44	0.004	−8	4	16	Caudate_L

table shows 3 local maxima more than 8.0 mm apart.

Height threshold: T = 3.2, p = 0.005 (1.000) {p<0.01 (unc.)}.

Extent threshold: k = 0 voxels p = 1.000 (1.000)).

Degrees of freedom = [1.0, 9.0].

**Table 2:** Clusters of face-selective activations in a Second level random-effects analysis for the localizer contrast (acquaintance faces>unfamiliar faces).

cluster p(cor)	cluster equiv k	voxel p(FDRcor)	voxel T	voxel p(unc)	x	y	z	
0.961	83	0.136	9.71	0.000	60	0	−18	Temporal_Mid_R
0.000	1756	0.136	7.74	0.000	0	42	8	Cingulum_Ant_L
		0.136	6.20	0.000	6	56	16	Frontal_Sup_Medial_R
		0.181	4.55	0.001	−2	26	16	Cingulum_Ant_L
0.356	206	0.136	6.05	0.000	52	−66	20	Temporal_Mid_R
		0.208	3.92	0.002	62	−54	12	Temporal_Mid_R
0.944	89	0.136	6.01	0.000	−44	22	2	Frontal_Inf_Tri_L
0.615	154	0.136	5.92	0.000	−8	−40	16	Cingulum_Post_L
		0.205	4.06	0.001	8	−38	18	Cingulum_Post_R
0.998	50	0.136	5.91	0.000	38	28	−18	Frontal_Inf_Orb_R
0.841	114	0.207	3.98	0.002	14	−6	22	Caudate_R
1.000	22	0.144	5.37	0.000	32	6	−20	Temporal_Pole_Sup_R
0.905	100	0.154	5.01	0.000	32	−28	−20	Fusiform_R
		0.220	3.13	0.006	32	−38	−18	Fusiform_R
0.993	62	0.195	4.25	0.001	2	−20	4	Thalamus_R
0.913	98	0.156	4.85	0.000	34	−12	−20	Hippocampus_R
0.992	63	0.205	4.08	0.001	−54	12	22	Frontal_Inf_Oper_L
		0.214	3.35	0.004	−48	4	22	Precentral_L
1.000	4	0.208	3.79	0.002	60	−14	−24	Temporal_Inf_R
1.000	33	0.208	3.70	0.002	−52	32	8	Frontal_Inf_Tri_L
0.998	52	0.208	3.62	0.003	−42	−58	16	Temporal_Mid_L
1.000	36	0.208	3.61	0.003	−14	6	14	Caudate_L
1.000	10	0.213	3.48	0.003	30	−40	−2	ParaHippocampal_R
0.999	43	0.214	3.34	0.004	2	−62	20	Calcarine_R
1.000	2	0.216	3.26	0.005	−34	−40	−4	ParaHippocampal_L
1.000	7	0.219	3.21	0.005	−60	−18	−10	Temporal_Mid_L

table shows 3 local maxima more than 8.0 mm apart.

Height threshold: T = 3.2, p = 0.005 (1.000) {p<0.005 (unc.)}.

Extent threshold: k = 0 voxels, p = 1.000 (1.000).

Degrees of freedom = [1.0, 9.0].

**Table 3:** Clusters of face-selective activations in a Second level random-effects analysis for the contrast newly-learned faces>unfamiliar faces).

cluster p(cor)	cluster equiv k	voxel p(FDRcor)	voxel T	voxel p(unc)	x	y	z	
0.290	247	0.616	5.86	0.000	−8	−38	18	Cingulum_Post_L
0.026	484	0.616	4.31	0.001	−16	10	10	Caudate_L
		0.616	3.58	0.003	−18	0	−4	Pallidum_L
1.000	14	0.616	4.68	0.001	−56	−52	14	Temporal_Mid_L
0.944	95	0.616	4.02	0.002	16	8	8	Caudate_R
0.956	90	0.616	3.93	0.002	12	−2	18	Caudate_R
		0.616	3.24	0.005	18	2	22	Caudate_R
1.000	41	0.616	3.86	0.002	30	−6	−6	Putamen_R
1.000	16	0.616	3.83	0.002	34	−12	−18	Hippocampus_R
1.000	27	0.616	3.31	0.005	−48	38	18	Frontal_Inf_Tri_L

table shows 3 local maxima more than 8.0 mm apart.

Height threshold: T = 3.2, p = 0.005 (1.000) {p<0.005 (unc.)}.

Extent threshold: k = 0 voxels, p = 1.000 (1.000).

Degrees of freedom = [1.0, 9.0].

### ROI-based analysis

**Figure 2:**
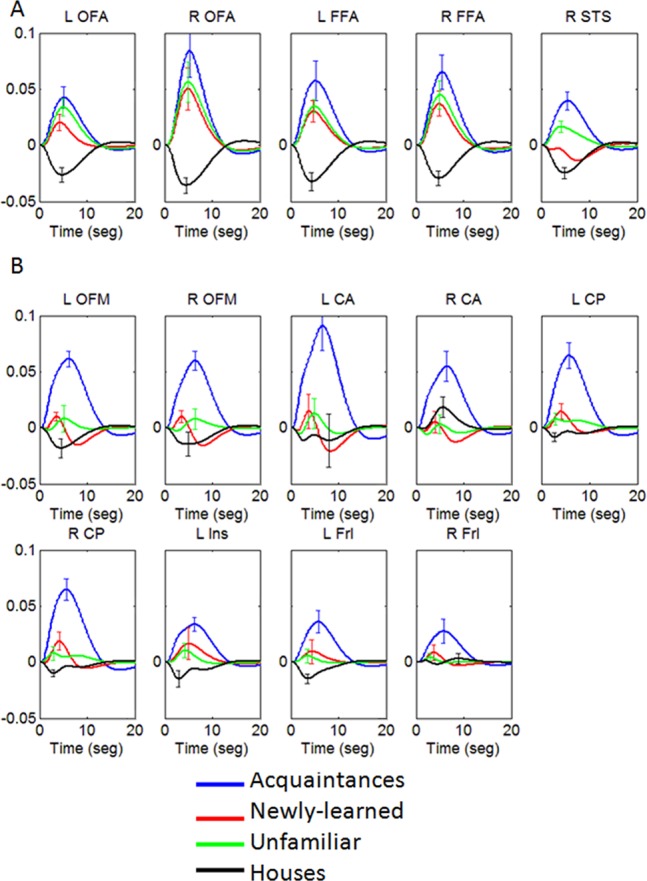
Mean HRFs average across subjects for different face conditions in the selected ROIs. Error bars denote the standard error of the mean.

The mean HRFs for each ROI are shown in Figure 2A. Within the ROIs belonging to the core system (OFA, FFA and pSTS), the HRF responses observed for all face conditions showed the typical positive ongoing pattern, except for the newly-learned response in pSTS, which were negative. These responses had a width of approximately 10 s. In all the extended system ROIs (Figure 2B), HRFs for faces of acquaintances were visibly larger than for unfamiliar and newly-learned faces. Also, in these ROIs the HRFs for faces of acquaintances were longer in duration than those related to other face conditions, more clearly so in the right mOF and right AC, where the HRF had a width of almost 15 s. The responses to newly-learned and unfamiliar faces seemed to be of low amplitude but still were larger than the response to houses in all ROIs (except right AC), although responses to newly-learned faces were somewhat larger in PC and Ins.

**Figure 3:**
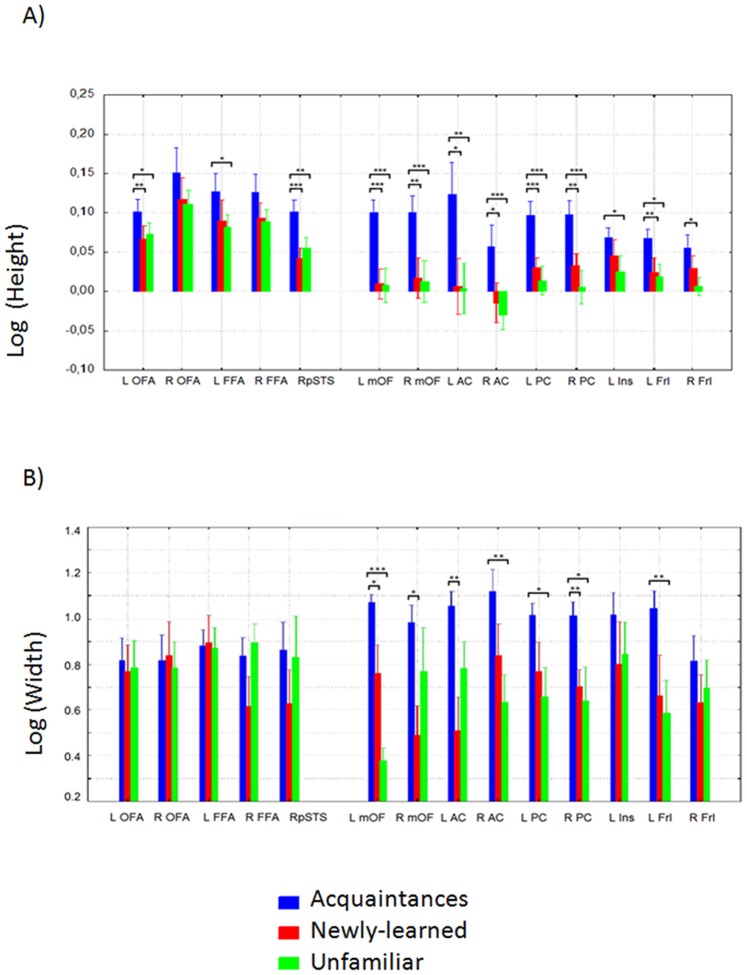
Bar graphs showing the parameters extracted from the estimated HRFs, for all face condition and all ROIs A) Height B) Width. Error bars correspond to the standard error. The results from the two-way interaction rm ANOVA between face condition and ROI are shown (significant differences between the conditions are indicated: * p<0.05, ** p<0.01, ***p<0.001).

The average height for each face condition across ROIs is displayed in Figure 3A. The corresponding rm-ANOVA (3 face conditions×14 ROIs) showed highly significant effects of Face condition (F(2,18) = 30.4, p<0.000002, H-F epsilon = 0.997, p<0.000002), and ROI (F(13,117) = 4.44, H-F epsilon  = 0.26, p<0.008), and a significant interaction between these two factors (F(26,234) = 2, p<0.005, H-F epsilon  = 0.5, p<0.03). Planned comparisons showed that this interaction was driven by the much larger responses to faces of acquaintances than to unfamiliar faces in the extended system (collapsing across ROIs of the extended system, F(1,9) = 55.24. p<0.00004), with a much smaller difference in the core ROIs (F(1,9) = 10.25.p<0.01) (see Figure S1 in File S1). The contrast for the interaction between lumped core vs. lumped extended ROIs on one hand, and faces of acquaintances vs. unfamiliar faces on the other, was significant (F(1,9) = 7.34, p<0.02). However the corresponding interactions involving acquaintances vs. newly-learned (F(1,9) = 1.63, n.s.) and unfamiliar vs. newly-learned (F(1,9) = 1.39, n.s.) were not significant. (See Table S1 in File S1 for other planned comparisons results).

The mean HRF widths for face condition against ROIs are displayed in Figure 3B. The corresponding rm-ANOVA (3 face conditions×14 ROIs) for width evinced a significant effect of Face condition (F(2,18) = 6.2, p<0.0089, H-F epsilon  = 1 p<0.0089), but not for ROI (F(1,9) = 0.62, n.s.). The interaction between these two factors was also significant (F(26,234) = 1.69, p<0.02, H-F epsilon  = 0.8, p<0.03). The contrast for the interaction between lumped core vs. lumped extended ROIs on one hand, and the three face conditions on the other, was significant (F(2,18) = 5.02, p<0.018). The interactions of ROI type (core vs. extended) and the faces of acquaintances vs. unfamiliar (F(1,9) = 9.58, p<0.01), and of ROI type with faces of acquaintances vs. newly-learned faces (F(1,9) = 6.24, p<0.034), were significant, whereas the same interaction with newly-learned vs. unfamiliar was not (F(1,9) = 0.41, n.s.). Within the lumped core ROIs, significant differences for width between pairs of face conditions were absent (all F(1,9)<1.63), whereas in the lumped extended system ROIs, the difference in width for both acquaintances vs. unfamiliar (F(1,9) = 17.9, p<0.0023) and acquaintances vs. newly-learned (F(1,9) = 24.8, p<0.0008) were highly significant. However, the contrast for newly-learned vs. unfamiliar was not (F(1,9) = 0.003, n.s.). (See Figure S2, and Table S2 in File S1 for other planned comparisons results.).

**Figure 4:**
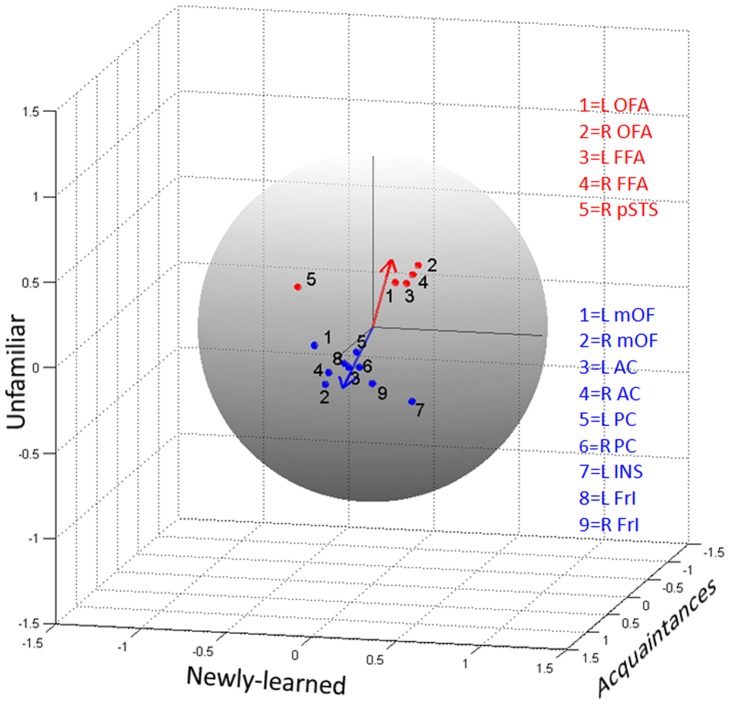
Activation profile in the tuning space. Each ROI is represented in the unitary sphere as a normalized three dimensional vector composed by the response to each face condition. The areas of the estimated HRF were used to describe the intensity of the response to each face condition. The core system ROIs are shown in red, while the extended system ROIs are in blue. The red vector is the mean profile of four ROIs of the core system (bilateral OFA and FFA). The blue vector is the mean profile of the extended system ROIs.

In order to view the tuning for face types more clearly, we defined an activation profile in each ROI as a unitary norm vector whose components corresponded to the area of the HRFs for each of the three face conditions (Figure 4). Therefore in these profiles, amplitude and duration measures were combined. As shown in the figure, these ROIs are grouped into two distinct clusters within the tuning space. One group of ROIs was comprised by the OFAs and FFAs, which exhibited little face type selectivity. The other group included the mOFs, ACs, PCs and FrI, characterized by large responses to faces of acquaintances and little response to other types of faces. Two ROIs do not fit into these clusters: the left insula, which responded to both faces of acquaintance and newly-learned faces; and the right pSTS, which responded to both familiar and unfamiliar faces, with negative response to newly-learned faces.

## Discussion

The amplitude and duration of BOLD responses to unfamiliar, visually-familiar, and personally-familiar faces were measured in several ROIs within the face processing system. Responses to unfamiliar faces were present in all core areas (OFA, FFA and pSTS), which also responded to visually and personally familiar faces. Only small differences in response amplitude and duration across face types were found in these ROIs. In contrast, most areas of the extended system (e.g. mOF) responded very weakly to unfamiliar and visually-familiar faces, but exhibited large and prolonged reactions to personally-familiar faces.

Before discussing the ROI data in more detail, we examine the consistency of our different voxelwise analyses with previously reported work. When faces were compared to non-face objects, both the FFA and the OFA were activated bilaterally as described in many studies [17]. Additionally, in the newly-learned>unfamiliar face contrast, we replicated the reported activations for PC, right hippocampus and left FrI [18], but not for OFA or FFA [19]. For the acquaintance>unfamiliar face contrast we also replicated the reported activations in pSTS, AC, PC, Ins, and mOF [20] but not in amygdala and middle temporal gyrus as reported in other studies [21]. Thus our voxelwise analyses were largely consistent with previous work.

Our results evinced two distinct patterns of tuning-to-familiarity, which mainly correspond to the core and the extended face ROIs respectively. Core areas responded strongly to all face categories, with weak face-category selectivity. In the right OFA and FFA the response was equivalent in amplitude across face type, whereas in the left OFA and FFA a small advantage was present for personally-familiar. Previous work is inconsistent about the effect of face familiarity on core system responses. In FFA, larger activations (relative to unfamiliar) have been reported for famous [20], personally familiar faces [22], and newly-learned faces [19]. However, decreased activations [18], and null effects have been also reported [23].

In a distinct pattern, all regions of the extended system clearly responded more to faces of acquaintances than to either unfamiliar faces or newly-learned faces, with the largest effects in mOF, AC, and PC. In fact, in these ROIs there was little response to the latter types of faces. This extreme tuning for faces of acquaintances is roughly consistent with, but cannot be clearly measured, in the traditional voxelwise contrasts presented here and reported previously. In the case of mOF, our results are more noteworthy given the difficulty of measuring the BOLD signal in this region due to susceptibility artifacts (which may explain why some studies did not find the effect in this region, e.g. [22]). Note that mere visual familiarity did not activate these extended system areas.

A few ROIs did not fit neatly into the two categories described above. Responses to newly-learned and personally familiar faces did not differ in amplitude in the Insula. The right pSTS exhibited negative responses to newly-learned faces (but still larger than the response to houses which we used as reference). Negative responses to newly-learned faces have been previously found in the intra-parietal sulcus [24], and in the amygdala [3], and could be related to inhibition that segregates these minimally familiar stimuli from those containing social information deserving further processing. Similar negative responses, have been also found for neutral pictures [8] and words [9] intermingled with emotional stimuli.

We also analyzed the duration of face-related activity in each ROI. This parameter has been ignored up to now in studies of face familiarity. The duration of the responses did not vary across face type in the core areas. However, in the extended system the responses to faces of acquaintances were apparently longer than those for the other types of face, and were also longer than in the core system. Both results suggest that personally-familiar faces are associated with prolonged neural processing. This different temporal dynamic should depend on both the information content offered by each kind of face and the position of the studied region within the visual hierarchy. Note that the core areas are conceived to be involved in a fast, feed-forward, and relatively automatic analysis of the visual cues afforded by faces [2], whereas the extended system includes areas involved in processes potentially prolonged in time (e.g. rumination and long lasting emotions in the mOF). However, we are cautious about this conclusion for several reasons. Responses to newly-learned and unfamiliar faces were very small in the extended system ROIs, entailing less reliable estimations of width. Furthermore, between-ROI differences in HRF width could be due to confounding factors such as regional differences in neurovascular coupling [25]. In this context, the results from the right PC are of special significance since its responses to all faces were large and statistically reliable. Since HRF width was still larger there for personally familiar faces despite invariant neurovascular coupling, longer neural processing seems a likely explanation for the effect.

To recapitulate, both amplitude tuning and HRF duration indicate a sharp dichotomy in the response pattern in the ROIs we studied here. The two measures were combined for the activation profile plot shown in Figure 4. The data could in principle be uniformly distributed over the whole sphere of possible functional profiles, with different clusters gradually blending into each other. However, the profiles were clustered into two clearly disjoint groups. Type I areas responded to all faces more or less equivalently, whereas type II areas were extremely tuned, presenting enhanced and prolonged responses only to faces of acquaintances. Importantly, type II areas were not responding to visual familiarity.

Type II profiles could be related to the retrieval of different types of memory, including two main candidates: on one hand semantic and episodic declarative memories, and on the other emotional associations. We cannot differentiate the relative contribution of these candidates in this study, although several lines of evidence suggest that emotional associations could be important. Emotional associations are unavoidable after close personal contact with other people. Accordingly, the subjects in the present study rated faces of acquaintances as very pleasant but newly-learned faces as neutral. We have reported that skin conductance responses (SCR) are larger for faces of acquaintances than for both unfamiliar faces and newly-learned faces [26]. Congruently, increased HRF duration has been described for affective pictures [8] or emotional words [9] relative to neutral stimuli in the frontal superior medial/AC and PC. In these studies the intensity of the emotional experience was directly related to HRF amplitude and duration [8].

The presence of type II profiles in the mOF is especially interesting and fits with a body of previously reported data. Lesions to the mOF abolish the enhanced SCRs for familiar faces [27]. This area exhibits a significant correlation between BOLD and SCR amplitudes [28]. Furthermore, the mOF is thought to play a role in representing the reward value of stimuli [29]. Congruently, the attractiveness or trustworthiness of faces is related to mOF activation [30]. Prolonged neural responses to emotional (compared with neutral) facial expressions have been found in the mOF with intracranial single unit recordings [31]. Therefore, the type II profile found in mOF is consistent with what is known about its role in the processing of emotional information.

On the other hand, the role of declarative memories in generating type II profiles cannot be excluded. A large store of semantic and episodic memories are also available for personal acquaintances. Anterior and medial temporal lobe structures have been implicated in the processing of declarative knowledge. Enhanced and prolonged neuronal unit activity to recognized- as compared to unrecognized-familiar faces has been found in medial temporal structures with intracranial recordings [32]. At least two approaches are available to dissect the roles of declarative memories and emotional associations in generating type II profiles. Firstly, one could broaden the profile analysis by including responses to artificially learned faces selectively associated to either declarative or affective information. Artificial learning of face affective information has been reported [33], and was related to increased activations in AC. Artificial learning of face related semantics has also been reported, coupled to increased activation of anterior temporal areas [34]. Thus, inclusion of these artificial stimuli in activation profiles, as well as measurement of the durations of the corresponding activations is necessary.

In an alternate approach, one can study activation profiles/temporal dynamics of different types of faces in brain damaged patients with dissociations of declarative and affective knowledge. This has been done for prosopagnosia, where the ability to overtly recognize faces is lost [35]. In some cases of this disorder, emotional memories are available for previously familiar faces as evinced by larger SCRs for familiar- than for unfamiliar-faces [36]. Interestingly, these cases can exhibit larger fMRI responses for personally-familiar than for unfamiliar faces in the extended system (e.g. PC and mOF) [13]. Face activation profiles must also be studied in the reverse dissociation (impaired emotional association and intact declarative knowledge for faces, e.g. Capgras syndrome [37]).

Some limitations of this study must be addressed. One problem is the low power due to the small number of subjects. Performing similar experiments with a larger number of subjects is necessary. Another problem, as pointed out by [38], is the potential difference between memories for faces artificially learned in the laboratory and those learned naturally in daily life. Therefore, use of this type of artificial stimuli should be complemented with that of real-life stimuli for which the amount of different kinds of knowledge can be gauged. This brings us to another limitation. We only included faces of close friends and family in the personally-familiar category. Consequently, all of these faces were rated as agreeable by all subjects. Artificially learned faces associated with different affective properties activate dissimilar brain regions [33]. In future studies of activation profiles, personally familiar faces of different valence (e.g. agreeable vs. disagreeable) should be included.

In conclusion, our results indicate that the neural processing of different types of familiar faces not only differs in degree, but is probably mediated by qualitatively distinct mechanisms. Several areas in the extended face system were virtually activated only by faces of personal acquaintances. These activations were longer lasting than those produced by mere visual familiarity. The short and spatially restricted response to visual familiarity is consistent with activity in a feed forward circuit [39]. On the other hand, the prolonged and spatially disseminated response to personal familiarity suggests a network incorporating multiple feed-forward routes operating in parallel, and very likely feedback circuitry [40]. To elucidate these candidate mechanisms, the activation profiles and temporal dynamics of face sensitive areas must be further studied.

## Supporting Information

File S1 Figure S1. Bar graphs showing the median of the height parameter of the core system ROIs(left and right OFA, left and right FFA, and right pSTS) and the extended ROIs (left and right PC, left and right AC, left and right mOF, left and right FrI and left Ins. Error bars corresponded to the standard error. Figure S2. Bar graphs showing the median of the width parameter of the core system ROIs(left and right OFA, left and right FFA, and right pSTS) and the extended ROIs (left and right PC, left and right AC, left and right mOF, left and right FrI and left Ins. Error bars corresponded to the standard error. Table S1. Results of the planned comparison in the rm-ANOVA over height values. Two factors: Face condition (3 levels) and ROI (14 levels). Table S2. Results of the planned comparison in the rm-ANOVA over width values. Two factors: Face condition (3 levels) and ROI (14 levels).
(DOC)
