# Supplementary material for: Timing and Tuning for Familiarity of Cortical Responses to Faces
Source: PLoS One. 2013 Oct 9;8(10):e76100. doi: 10.1371/journal.pone.0076100 (PMC3794035; doi:10.1371/journal.pone.0076100)
Supplement: File S1 — Figure S1. Bar graphs showing the median of the height parameter of the core system ROIs(left and right OFA, left and right FFA, and right pSTS) and the extended ROIs (left and right PC, left and right AC, left and right mOF, left and right FrI and left Ins. Error bars corresponded to the standard error. Figure S2. Bar graphs showing the median of the width parameter of the core system ROIs(left and right OFA, left and right FFA, and right pSTS) and the extended ROIs (left and right PC, left and right AC, left and right mOF, left and right FrI and left Ins. Error bars corresponded to the standard error. Table S1. Results of the planned comparison in the rm-ANOVA over height values. Two factors: Face condition (3 levels) and ROI (14 levels). Table S2. Results of the planned comparison in the rm-ANOVA over width values. Two factors: Face condition (3 levels) and ROI (14 levels). (DOC) [file pone.0076100.s001.doc]

## Supplementary material

## Timing and tuning for familiarity of cortical responses to faces.

Maria A. Bobes*, Agustin Lage*, Ileana Quiñones**, Lorna García**, and Mitchell Valdes-Sosa*.

Figure S1. Bar graphs showing the median of the height parameter of the core system ROIs( left and right OFA, left and right FFA, and right pSTS) and the extended ROIs (left and right PC, left and right AC, left and right mOF, left and right FrI and left Ins. Error bars corresponded to the standard error.


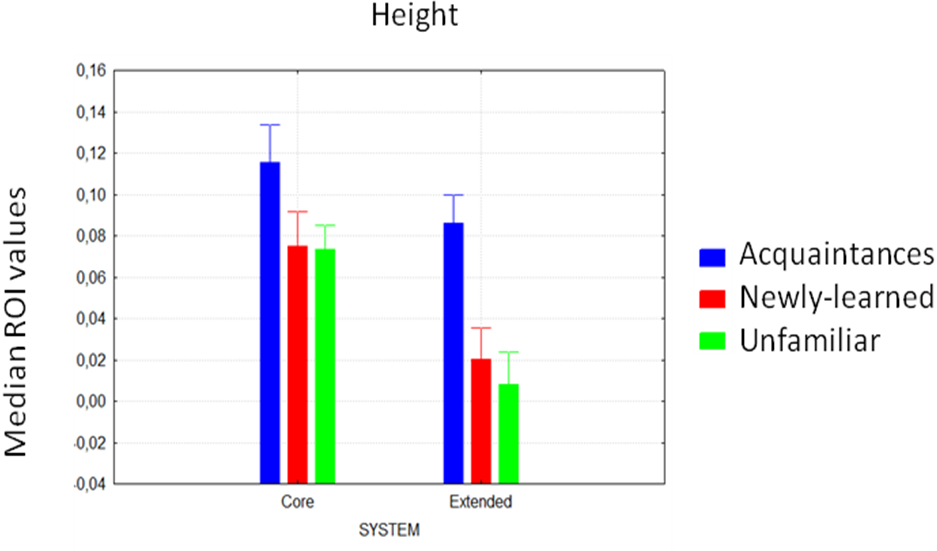


Figure S2. Bar graphs showing the median of the width parameter of the core system ROIs( left and right OFA, left and right FFA, and right pSTS) and the extended ROIs (left and right PC, left and right AC, left and right mOF, left and right FrI and left Ins. Error bars corresponded to the standard error.


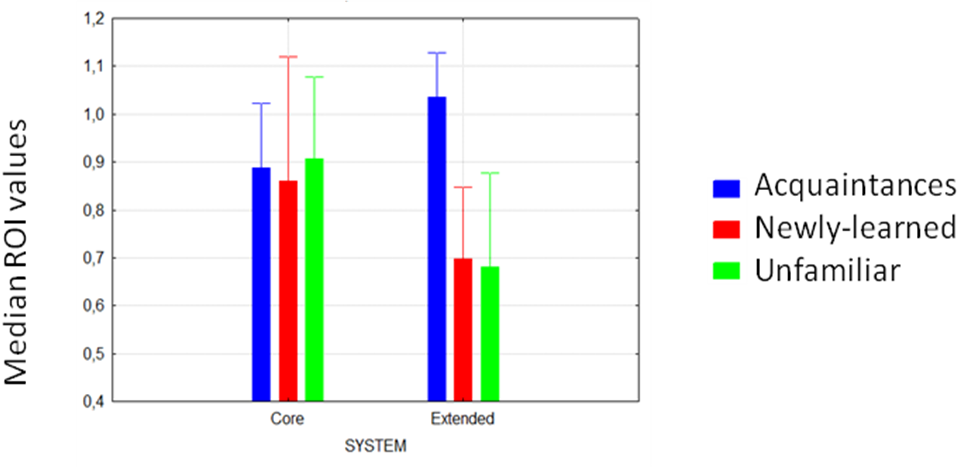


Table S1: Results of the planned comparison in the rm-ANOVA over height values . Two factors: Face condition (3 levels) and ROI (14 levels)

| Contrast | All ROIs | Core system | Extended system |
| --- | --- | --- | --- |
|  |  | Left OFA Right OFA | Left mOF right mOF, Left CA Right CA |
|  |  | Left FFA Right FFA, Right pSTS | Left CP Right CP, Left Ins, Left FrI Right FrI |
| Acquaintances vs. unfamiliar faces: | F(1,9)=51.8 | F(1,9)=10.71 | F(1,9)=52.24 |
|  | p<0.0001 | p<0.009 | P<0.0001 |
| Acquaintances vs. newly-learned faces | F(1,9)= 48.5 | F(1,9)=11.55 | F(1,9)=34.33 |
|  | P<0.0001 | p<0.007 | P<0.0002 |
| Newly-learned faces vs.unfamiliar faces | F(1,9)= 0.6 | F(1,9)= 0.0007 | F(1,9)= 1.03 |
|  | p<0.02 | p<0.97 | p<0.33 |

| Contrast | Left OFA | Right OFA | Left FFA | Right FFA | Right STS | Left mOF | Right mOF | Left AC | Right AC | Left PC | Right PC | Left INS | Left FrI | Right FrI |
| --- | --- | --- | --- | --- | --- | --- | --- | --- | --- | --- | --- | --- | --- | --- |
| Acquaintances vs 0 | F=41,441 p<0,000 | F=22,032 p<0,001 | F=29,633 p<0,000 | F=31,382 p<0,000 | F=49,254 p<0,000 | F=39,626 p<0,000 | F=21,814 p<0,001 | F=8,950 p<0,015 | F=5,086 p<0,05 | F=29,109 p<0,000 | F=30,194 p<0,000 | F=30,528 p<0,000 | F=34,830 p<0,000 | F=11,714 p<0,008 |
| Newly-Learned vs 0 | F=16,130 p<0,003 | F=18,838 p<0,002 | F=11,297 p<0,008 | F=23,116 p<0,001 | F=9,518 p<0,013 | F=0,255 p<0,626 | F=0,454 p<0,517 | F=0,031 p<0,865 | F=0,342 p<0,573 | F=5,459 p<0,044 | F=4,983 p<0,051 | F=5,086 p<0,05 | F=1,823 p<0,210 | F=3,281 p<0,104 |
| Unfamiliar faces vs 0 | F=25,710 p<0,001 | F=42,209 p<0,000 | F=27,786 p<0,001 | F=33,394 p<0,000 | F=19,471 p<0,002 | F=0,135 p<0,722 | F=0,228 p<0,645 | F=0,014 p<0,909 | F=2,679 p<0,136 | F=0,608 p<0,456 | F=0,060 p<0,811 | F=1,541 p<0,246 | F=1,375 p<0,271 | F=0,313 p<0,589 |
| Acquaintances vs Unfamiliar faces | F=5,220 p<0,048 | F=4,486 p<0,063 | F=7,527 p<0,023 | F=4,937 p<0,053 | F=13,512 p<0,005 | F=31,745 p<0,000 | F=32,024 p<0,000 | F=19,11 p<0,002 | F=27,402 p<0,001 | F=24,554 p<0,001 | F=25,002 p<0,001 | F=5,825 p<0,039 | F=9,379 p<0,014 | F=5,039 p<0,051 |
| Newly-Learned vs Unfamiliar faces | F=0,303 p<0,595 | F=0,105 p<0,753 | F=0,291 p<0,603 | F=0,132 p<0,725 | F=0,850 p<0,381 | F=0,008 p<0,930 | F=0,159 p<0,699 | F=0,007 p<0,937 | F=0,367 p<0,560 | F=0,801 p<0,394 | F=2,082 p<0,183 | F=0,823 p<0,388 | F=0,219 p<0,651 | F=1,162 p<0,309 |
| Acquaintances vs Newly-Learned | F=11,479 p<0,008 | F=1,980 p<0,193 | F=3,823 p<0,082 | F=3,231 p<0,106 | F=25,331 p<0,001 | F=16,275 p<0,001 | F=18,525 p<0,002 | F=9,634 p<0,013 | F=5,399 p<0,045 | F=20,743 p<0,001 | F=18,173 p<0,002 | F=2,007 p<0,190 | F=11,717 p<0,008 | F=7,950 p<0,020 |

Table S2 Results of the planned comparison in the rm-ANOVA over width values . Two factors: Face condition (3 levels) and ROI (14 levels)

|  | All ROIs | Core system | Extended system |
| --- | --- | --- | --- |
|  |  | Left OFA Right OFA | Left mOF right mOF, Left CA Right CA |
|  |  | Left FFA Right FFA, Right pSTS | Left CP Right CP, Left Ins, Left FrI Right FrI |
| Acquaintances vs. unfamiliar faces: | F(1,9)=6.74 | F(1,9)= 0.006 | F(1,9)=124.8 |
|  | p< 0.028 | p< 0.94 | p<0.0008 |
| Acquaintances vs. newly-learned faces | F(1,9)=10.7 | F(1,9)= 0.59 | F(1,9)= 18.0 |
|  | p< 0.009 | p< 0.46 | p<0.002 |
| Newly-learned faces vs.unfamiliar faces | F(1,9)= 0.27 | F(1,9)= 1.63 | F(1,9)= 0.003 |
|  | p< 0.61 | p< 0.23 | p< 0.95 |

| Contrast | Left OFA | Right OFA | Left FFA | Right FFA | Right STS | Left mOF | Right mOF | Left AC | Right AC | Left PC | Right PC | Left INS | Left FrI | Right FrI |
| --- | --- | --- | --- | --- | --- | --- | --- | --- | --- | --- | --- | --- | --- | --- |
| Acquaintances vs Unfamiliar faces | F=0,029 p<0,868 | F=0,022 p<0,884 | F=0,004 p<0,94 | F=0,170 p<0,689 | F=0,023 p<0,881 | F=19,356 p<0,000 | F=0,91 p<0,361 | F=4,068 p<0,074 | F=12,407 p<0,006 | F=5,782 p<0,039 | F=5,499 p<0,043 | F=1,052 p<0,457 | F=12,14 p<0,006 | F=0,377 p<0,553 |
| Newly-Learned vs Unfamiliar faces | F=0,016 p<0,902 | F=0,171 p<0,689 | F=0,038 p<0,850 | F=6,384 p<0,032 | F=0,744 p<0,411 | F=8,301 p<0,018 | F=2,717 p<0,134 | F=1,517 p<0,249 | F=1,242 p<0,293 | F=0,284 p<0,607 | F=0,136 p<0,721 | F=0,029 p<0,869 | F=0,098 p<0,761 | F=0,095 p<0,765 |
| Acquaintances vs Newly-Learned | F=1,152 p<0,706 | F=0,017 p<0,897 | F=0,012 p<0,913 | F=1,708 p<0,223 | F=2,015 p<0,189 | F=7,154 p<0,025 | F=8,511 p<0,017 | F=10,75 p<0,009 | F=3,54 p<0,09 | F=3,626 p<0,089 | F=13,144 p<0,005 | F=0,733 p<0,413 | F=2,747 p<0,131 | F=1,715 p<0,218 |

|  | Left mOF | Right mOF | Left CA | Right CA | Left CP | Right CP | Left INS | Left FrI | Right FrI |
| --- | --- | --- | --- | --- | --- | --- | --- | --- | --- |
| Acquaintances Core (L OFA, Right OFA,L FFA, Right FFA) vs | F=11.54 p<0,007 | F=1,938 p<0,19 | F=4,95 p<0,05 | F=8,46 p<0,01 | F=7.91 p<0,02 | F=36.11 p<0,03 | F=4,49 p<0,06 | F=8,77 p<0,01 | F=0,05 p<0,84 |
